# Spectroscopic Optical Coherence Tomography for Thin Layer and Foil Measurements

**DOI:** 10.3390/s20195653

**Published:** 2020-10-02

**Authors:** Aleksandra M. Kamińska, Marcin R. Strąkowski, Jerzy Pluciński

**Affiliations:** Department of Metrology and Optoelectronics, Faculty of Electronics, Telecommunications and Informatics, Gdańsk University of Technology, 11/12 Gabriela Narutowicza St., 80-233 Gdańsk, Poland; aleksandra.kaminska@pg.edu.pl (A.M.K.); marcin.strakowski@pg.edu.pl (M.R.S.)

**Keywords:** optical coherence tomography, thin layer, thin film, thin foil, Fabry–Pérot interferometer

## Abstract

The main goal of this research was to assess if it is possible to evaluate the thickness of thin layers (both thin films on the surface and thin layers below the surface of the tested object) and foils using optical coherence tomography (OCT) for thickness assessment under the resolution of the standard commercially available OCT measurement system. In the proposed solution, light backscattered from the evaluated thin layer has been expressed as a multiple beam interference. Therefore, the OCT system was modeled as a two-beam interferometer (e.g., Michelson), in which one beam propagates from the reference arm and the other comes from a Fabry–Pérot interferometer. As a consequence, the mathematical model consists of the main Michelson interferometer, in which the measuring arm represents the Fabry–Pérot interferometer. The parameters of the layer (or foil) are evaluated by analyzing the minimum value of the interference contrast. The model developed predicts the behavior of the thin layers made from different materials (with different refractive indexes) with different thickness and located at different depths. To verify the correctness of the proposed model, an experiment with a wedge cell has been carried out. The wedge cell was shifted across the scanning beam using a linear translation stage with a micrometer screw under the scanning head. The relationship between the thickness of the gap of the wedge cell and the OCT output signal is presented. For the additional verification of the proposed model, the results of the measurements of the thickness of the thin foil were compared with the theoretical results of the simulations. The film thickness was evaluated based on the calculated positions of the minimum value of interference contrast. A combination of the standard potentialities of OCT with the proposed approach to analyzing the signal produces new metrological possibilities. The method developed allows us to evaluate thickness under the resolution of the system and the location of the layer as well. This produces the possibility of measuring a layer which is covered by another layer. Moreover, it is possible to create a thickness map with high sensitivity to thickness changes. These experiments and simulations are the culmination of preliminary research for evaluating the potential of the proposed measurement method.

## 1. Introduction

Optical coherence tomography (OCT) is an optical measurement technique for the visualization of the internal structure of a tested object. By measuring optical backscattering in a cross-sectional plane through the sample, it is possible to obtain high-resolution 2D and 3D tomography images [1,2,3,4]. An additional advantage of OCT is its non-contact and non-destructive character. Due to this, OCT is widely applied in medicine, especially in ophthalmology [5,6]. Moreover, OCT has applications in biology [7,8], nanotechnology [9,10], and material science for the examination of optically scattering materials [11,12] (including human tissue, polymers, ceramics). The rapid development of OCT has caused the rise of many derivative OCT imaging modalities such as Doppler OCT (D-OCT), polarization-sensitive OCT (PS-OCT), and spectroscopic OCT (S-OCT), which delivers additional information about the tested samples [13,14,15,16]. Apart from information about the location of scattering centers, these methods also deliver information about the optical properties of the sample. In this work, we propose a new functional OCT that is dedicated to the measurement of thin layers. This method allows for the measurement of thickness and the refractive index of thin layers, as well as the determination of its location beneath the surface of the tested sample. Additionally, it is possible to evaluate the properties of the layer which is covered by another layer.

Thin layer technologies have found widespread application, which includes the field of microelectronics [17,18] (e.g., for the production of quantum wells for quantum cascade lasers (QCLs) or layers in tunnel diodes), in optics [19] (as antireflection layers and for production elements in integrated optics), and in sensors of physical and chemical quantities [20]. Due to the rapid implementation of thin layer technology in many fields of science and industry, there are now opportunities available to develop and adopt the new fast and cheap measurement methods in order to assess the quality of manufactured products during production. For this purpose, it is necessary to be able to measure the parameters of thin layers, such as thickness, refractive index, homogeneity, and roughness. Due to the slight thickness of the layers, measurement techniques must be characterized by high resolution and repeatability. As a contactless, non-destructive, and fast method, OCT has become an interesting candidate for that purpose. In scientific research and technology, one may find many attempts to use OCT for the evaluation of layered structures. The research described by Muhammad Faison Shiraz et al. [21] may be a good example of that. It utilized the spectral-domain optical coherence tomography system (SD-OCT system) with parallel scanning. As a result, an inline quality testing system for liquid crystal display (LCD) evaluation has been built. As well as possessing the ability to rapidly scan a large area, the system has a 5-µm axial resolution, which, according to the authors, is good enough to assess the quality of the protective layers of the LCD. Another application of OCT for layer evaluation is linked to the investigation of drug coatings. As an example, the uniformity and thickness of the coating were evaluated within a few micrometers resolution by the use of the standard SD-OCT system, which has been reported in [22,23]. Ultra-high-resolution OCT (UHR-OCT) with an axial resolution which is better and in some cases far better than 5 µm or is used in measurements for both medical (e.g., [24,25], especially in ophthalmology [26,27,28,29]) and non-medical purposes. One example of the use of UHR-OCT outside of medicine is the test of works of art. Chi Shing Cheung et al. used UHR-OCT to obtain “virtual” cross-section images of thin varnish layers [30] and Alessandra Vichi et al. used it to detect metal soaps in paintings [31]. Jakub Czajkowski et al. [32] have shown that ultra-high resolution OCT (UHR-OCT) can be utilized in printed electronics. In the paper, they presented measurements of layers of an antenna as a sample of multilayer and multi-material structures with a resolution of a few micrometers. The component contains five wires covered with an insulating layer. In other studies, UHR-OCT was used for printed electronics where Jakub Czajkowski et al. obtained the axial resolution better than 1 µm for measurements of a thin layer of the parylene C polymer [33].

Although the OCT method may be used to evaluate the layered structures, to date, there has not been sufficient research concerning thin layer (or foil) measurements below the standard OCT axial resolution, especially in the submicrometer regime. For standard commercially available OCT systems, such a thin layer would be expressed as a single line at OCT cross-sectional images. However, the motivation for conducting the research is supported by the fact that the optically transparent or semitransparent thin layers may affect the spectral characteristics of the backscattered light as a product of the interference of light reflected by the top and bottom surface of the layer similarly which occurs in a Fabry–Pérot interferometer. Promising results were reached by applying optical reflectometry. This concept was evaluated in the field of tear film measurements, which has been reported in [34]. Hui Lu et al. presented the optical reflectometer, which allowed for film thickness measurements with an accuracy at a level of 12 nm. Despite the high resolution and accuracy of the measurement, the optical reflectometry technique is only limited to the films at the surface of the tested object. Also, at her Ph.D. dissertation [35], Hui Lu presented an optical coherence tomography system integrated with optical reflectometry, which combined the high accuracy measurements of the film thickness with OCT imaging. She also observed the autocorrelation term at the OCT signal, however, it has not been used to evaluate the layers beneath the surface of tested objects.

The optical spectrum changes, related to the autocorrelation terms of the signal [36], can be measured by one of the functional types of OCT methods, which is called spectroscopic optical coherence tomography (S-OCT) and has been described in [10,37,38,39,40,41]. This concept, with the aid of time-frequency analysis of the OCT signal, was studied by us and reported in [10]. Time-frequency analysis has been applied to retrieve the signal backscattered from a particular layer and estimate its thickness changes. Afterward, this method was investigated by Valentin Aranha dos Santos et al., where the authors presented a deep analysis of this phenomenon [42]. The usability of the method was expressed by measuring the thin photoresist film on the silicon substrate. The capability of retrieving spectral information combined with other OCT features, which are spatially resolved measurements, is leading to a new method for evaluating the properties of thin films. The presented scientific report includes a comprehensive study of OCT signal analysis to estimate the thickness of the thin film, as well as expressing the usability of the method for evaluating the thin layers beneath the sample surface.

## 2. Mathematical Modeling

**Figure 1:**
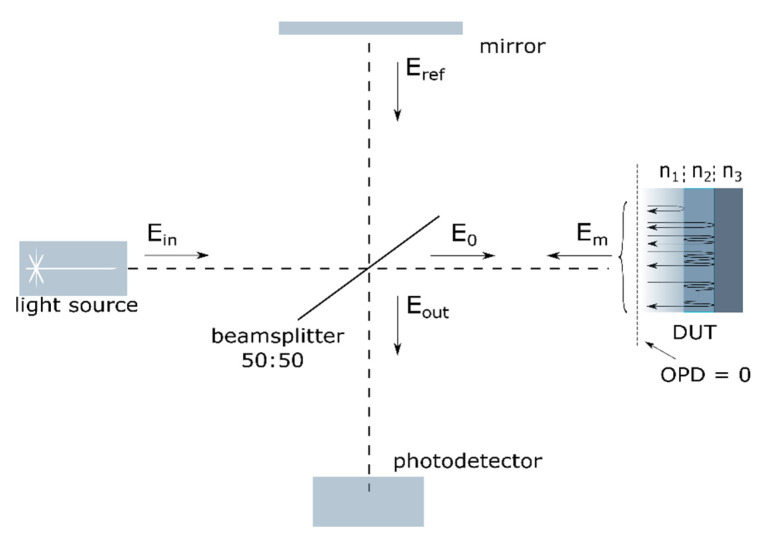
The principle of operation of the optical coherence tomography (OCT) system model. The raw laser beam enters the optical system through the beamsplitter, which separates an incoming wave into a reference and measurement arm. The reference beam is produced by reflection from the mirror, and the measurement beam is sourced from the Fabry–Pérot interferometer. The device under test (DUT) is a thin film with a refractive index $n_{2}$, the medium above the film with refractive index $n_{1}$, and a substrate with refractive index $n_{3}$.

In general, a standard approach to the theory of optical coherence tomography is based on the estimation of the intensity of backscattered light from the particle point inside the evaluated object. For the frequency domain optical coherence tomography (FD-OCT), the capability for spatially resolved measurements is reached by the analysis of the frequency of interference pattern in the spectral domain. It means that if the optical path difference (OPD) between interfering beams is short enough, the observed spectra of the interference product can be modulated with low frequency. However, if the OPD is increased, the frequency would also be higher. Such a modulated pattern can be produced by the interference of the optical beams from measurement ($E_{m}$) and reference ($E_{\mathrm{ref}}$) arms of the interferometer, as shown in Figure 1. However, this signal can be influenced by another source of the interference signal, which is the interference between two or more beams backscattered from a different scattering point and guided together through the measurement arm.

During this study, we will show that for thin films with the optical thickness below the length of the coherence path of the source, the interference product from the film has an impact on the cross-correlation term of the recorded OCT measurement signal. The analysis requires the mathematical model, in which the principles of OCT and multibeam interactions are included.

The developed mathematical model of measurement systems is based on the wave theory of optics. In this approach, propagation of light through the thin layer was simulated inclusive of the reflection of light in the Fabry–Pérot interferometer, while the measurement system was modeled by a Michelson interferometer (see Figure 1). As a result, the proposed model presents the interference of multiple reflected waves in a sample and interference with the wave in reference arm in the Michelson interferometer.

### 2.1. Reflection of Scanning Beam from the Thin Layer

The OCT systems use the Gaussian beam as a scanning beam. In general, accurate analysis of the reflection of such a beam from the layer differs from the classic analysis of the reflection of a planar wave—it requires, e.g., taking into account the curvature of the wavefront and the Gouy effect [43,44,45]. The case, where the layer thickness is much smaller than the Rayleigh range of the scanning beam, is analyzed below (in typical OCT systems the Rayleigh range is in the order of several dozen or several hundred micrometers, and the reflection from a layer thickness of 10 μm or less is analyzed). Furthermore, it was assumed that the waist of the scanning beam is located at a distance from the layer which is much smaller than the Rayleigh range (such a condition is required to obtain a large lateral resolution of the OCT system). With these assumptions, the effects associated with the Gouy effect and the wavefront curvature may be neglected. In this particular analysis, it was also assumed that the scanning beam falls perpendicular to the measured layer.

**Figure 2:**
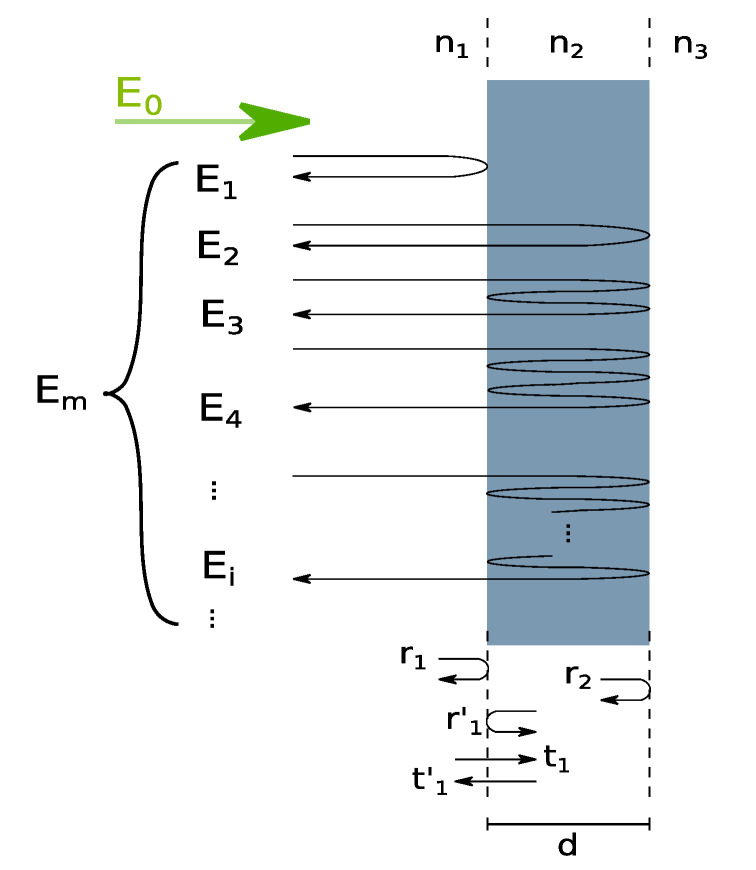
The propagation of the light in a thin layer, where $E_{0}$ and $E_{m}$ are complex amplitudes of the incident and reflected beams, respectively. $E_{m}$ equals the sum of complex amplitudes of the reflected waves $E_{1}$, $E_{2}$, …, $E_{i}$, … from the thin layer. The amplitudes $E_{i}$ depend on the transmission coefficients $t_{1}$ and ${t^{\prime }}_{1}$ and reflection coefficients $r_{1}$, ${r^{\prime }}_{1}$, and $r_{2}$. These coefficients depend on the layer thickness *d*, the refractive index of the layer $n_{2}$, and the refractive indexes of the media in front and behind the layer: $n_{1}$ and $n_{3}$.

The analysis is based on multiple reflections [44,45] on the interfaces between media with different refractive indices—see Figure 2.

The complex amplitude $E_{m}$ of the reflected beam from the layer can be presented as the sum of the infinite number of the complex amplitudes $E_{1}$, $E_{2}$, … of the reflected waves on the interfaces between different optical media:

(1) $$E_{m}=\overset{\infty }{\underset{i\;=\;1}{\sum }}E_{i},$$

where:

(2) $$E_{i}=\;\{\begin{array}{ll} r_{1}E_{0} & ,\;\mathrm{if}\;i\;=\;1\\ {(r^{\prime }}_{1}{)}^{i–2}r_{2}^{i–1}t_{1}{t^{\prime }}_{1}{\exp[–j4\pi (i\;–\;1)n}_{2}{d/\lambda ]E}_{0} & ,\;\mathrm{if}\;i\;>\;1 \end{array},$$

$r_{1}$, ${r^{\prime }}_{1}$, and $r_{2}$ are the reflection coefficients, $t_{1}$ and ${t^{\prime }}_{1}$ are the transmission coefficients (see Figure 2), *d* and $n_{2}$ are the thickness and the refractive index of the layer, respectively, $E_{0}$ is the complex amplitude of the incident beam, and *λ* is the wavelength in a vacuum (we assume that the layer is thin enough to neglect the effects of absorption within the layer and phenomena associated with possible anisotropy of the layer).

Based on the Fresnel equations:

(3) $$r_{1\;}=\;\frac{n_{1\;}{-\;n}_{2}}{n_{1\;}{+\;n}_{2}},$$

(4) $${r^{\prime }}_{1\;}=\;\frac{n_{2\;}{-\;n}_{1}}{n_{1\;}{+\;n}_{2}}{\;=\;-r}_{1},$$

(5) $$r_{2\;}=\;\frac{n_{2\;}{-\;n}_{3}}{n_{2\;}{+\;n}_{3}},$$

(6) $$t_{1\;}{=\;1\;+\;r}_{1\;},$$

(7) $${t^{\prime }}_{1\;}{=\;1\;+\;r^{\prime }}_{1\;}{=\;1\;-\;r}_{1},$$

where $n_{1}$ and $n_{3}$ are the refractive indexes (RI) of the media in front and behind the layer and $n_{2}$ is the RI of the layer, respectively, we can rewrite Equation (2) as:

(8) $$E_{i}\;=\;\{\begin{array}{ll} r_{1}E_{0} & ,\;\mathrm{if}\;i\;=\;1\\ {{(-r}_{1})}^{i-2}r_{2}^{i-1}{(1\;-\;r}_{1}^{2}{)\exp[-j4\pi (i\;-\;1)n}_{2}{d/\lambda ]E}_{0} & ,\;\mathrm{if}\;i\;>\;1 \end{array},$$

As the amplitudes, $E_{i}$ forms a geometric progression for *i* > 1, from Equation (8) we finally obtain:

(9) $$E_{m}\;=\;\left[r_{1}+\frac{{(1\;-\;r}_{1}^{2}{)r}_{2}{\exp[-j4\pi n}_{2}d/\lambda ]}{{1\;+\;r}_{1}r_{2}{\exp[-j4\pi n}_{2}d/\lambda ]}\right]E_{0}.$$

**Figure 3:**
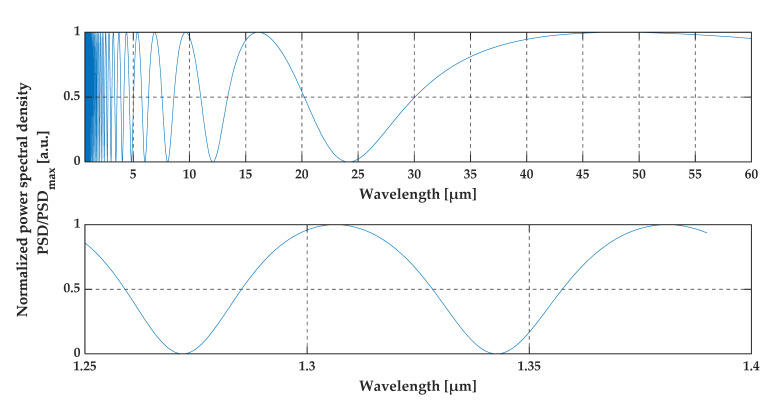
Interference signal obtained from a Fabry–Pérot etalon. A thin foil of diamond with a thickness of 5 µm was used as a cavity. Both plots present the spectral characteristics of the optical radiation (with a uniform spectral distribution) from the Fabry–Pérot etalon.

The OCT system measures the intensity of the interference signal, therefore, based on $E_{m}$ (Equation (9)), the power spectral density (PSD) of the back-reflected light from the thin layer was calculated and plotted in Figure 3. The upper chart shows the PSD of the interference signal over a wide spectral range, while the other one is limited to the range of standard OCT systems.

The intensity is a non-periodic function of wavelength (see Figure 3). The oscillatory response occurs over a wide spectrum. The rate of change of amplitude increases for shorter wavelengths. For this reason, we expect to observe the variable oscillation signal for the spectral range used in OCT systems.

### 2.2. Interference of Optical Beams from a Thin Layer and the Reference Arm in the OCT System

The intensity $I_{\mathrm{out}}$ of the output beam from the Michelson interferometer of the OCT system is equal to the average square of the module of the sum of the complex amplitudes ${E^{\prime }}_{m}$ and ${E^{\prime }}_{r}$ of beams coming from the measurement and reference arms and guided by the beamsplitter to the detector:

(10) $$I_{\mathrm{out}}(\lambda )\;=\;{\left|{E^{\prime }}_{m}{\;+\;E^{\prime }}_{r}\right|}^{2}.$$

For the 50:50 beamsplitter and lossless system using Equation (9), we can rewrite Equation (10) as

(11) $$I_{\mathrm{out}}(\lambda )\;=\;\frac{1}{4}{\left|r_{1}+\frac{{(1\;-\;r}_{1}^{2}{)r}_{2}{\exp[-j4\pi n}_{2}d/\lambda ]}{{1\;+\;r}_{1}r_{2}{\exp[-j4\pi n}_{2}d/\lambda ]}\;+\;\cos(2\pi \Delta z/\lambda )\right|}^{2}I_{\mathrm{in}}(\lambda ),$$

where Δ*z* is the difference in the length of the optical paths of the interfering beams at the output of the OCT system resulting from the difference between the distance between the distance of the measurement layer and the mirror in the reference arm from the beamsplitter, and $I_{\mathrm{in}}$ is the intensity of the input beam directed from the laser to the beamsplitter.

The model shows the relationship between the layer parameters (like the layer thickness (*d*), the refractive indexes ($n_{1}$, $n_{2}$, and $n_{3}$), the depth *z* at which the layer appears) and the output OCT signal in the wavelength (spectral) domain. The parameters assumed for the modeled system are summarized in Table 1.

**Figure 4:**
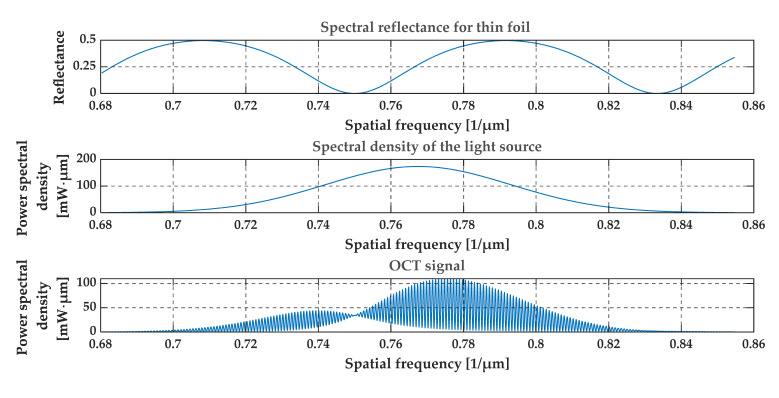
Theoretical spectral characteristics obtained from a thin foil of diamond. The thickness of the foil is 2.5 μm and the depth of the layer is 0.25 mm. Refractive indexes are $n_{1\;}=\;1,\;n_{2\;}=\;2.4,\;n_{3\;}=\;1.$

**Figure 5:**
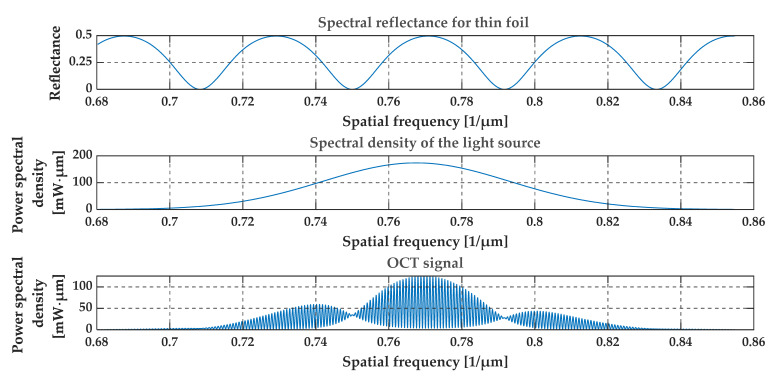
Theoretical spectral characteristics obtained from a thin foil of diamond. The thickness of the foil is 5 μm and the depth of the layer is 0.25 mm. Refractive indexes are $n_{1\;}=\;1,\;n_{2\;}=\;2.4,\;n_{3\;}=\;1.$

**Figure 6:**
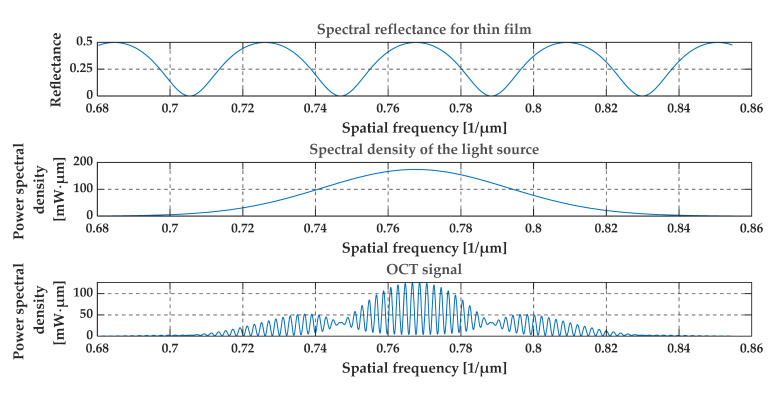
Theoretical spectral characteristics obtained from a thin film of diamond. The thickness of the layer is 5 μm and the depth of the layer is 0.1 mm. Refractive indexes are $n_{1\;}=\;1,\;n_{2\;}=\;2.4,\;n_{3\;}=\;1.$

**Figure 7:**
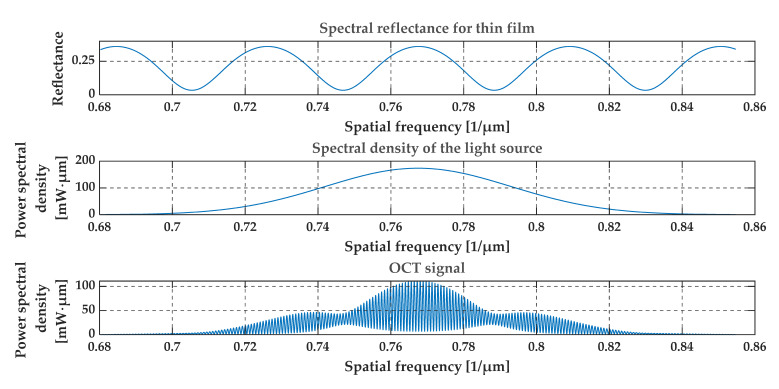
Theoretical spectral characteristics obtained from a thin film of diamond. The thickness of the film is 5 μm and the depth of the layer is 0.25 mm. Refractive indexes are $n_{1\;}=\;1,\;n_{2\;}=\;2.4,\;n_{3\;}=\;1.45.$

Figure 4, Figure 5, Figure 6 and Figure 7 present a simulation with theoretical curves of interference signal obtained from a thin film or foil, the power spectral density of the light source, and the output OCT signal for different parameters of the thin film or foil in a spatial frequency domain (i.e., in 1/*λ* domain). Figure 4 and Figure 5 consider a thin diamond foil. In the first case (Figure 4), the thickness of the layer is equal to 2.5 μm, and in the next case (Figure 5), the thickness is equal to 5 μm. Both values are under the theoretical resolution of the system at a given spectral range. Figure 5 and Figure 6 present the case of the thin diamond film at different depths (the depth is measured from the position, where OPD = 0—see Figure 1, i.e., from the position for which the difference in the length of the optical paths of the interfering beams from the arms of the interferometer equals zero). The simulation presents the change in the difference in the length of the optical paths of the interfering beams. Depending on the position of the thin film, the high-frequency signal differs. The last example (Figure 7) shows the influence of the refractive indexes of the surrounding media. The ratio $n_{1}$/$n_{3}$ is related to the depth modulation of the output OCT signal.

**Figure 8:**
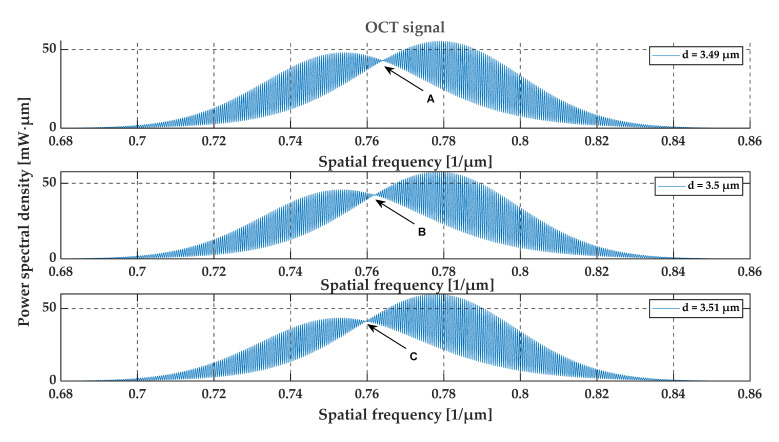
The relationship between the location of the local minimum of the interference contrast and the glass foil thickness. The arrows (A, B, C) show the discussed points.

**Figure 9:**
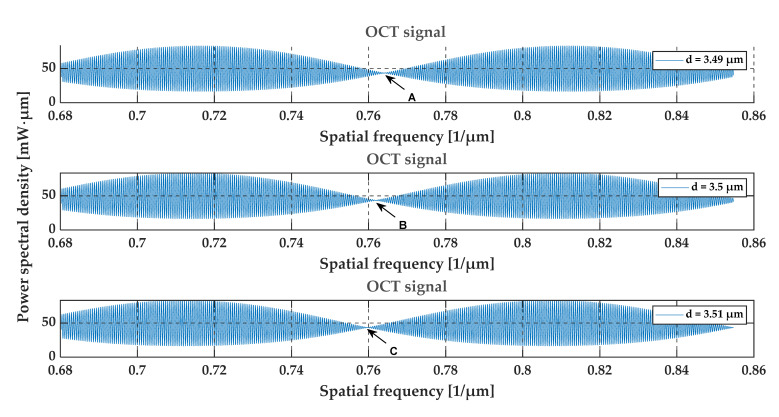
The OCT signal for glass foils for the uniform spectral characteristic of the light source. The arrows (A, B, C) show the discussed points.

**Figure 10:**
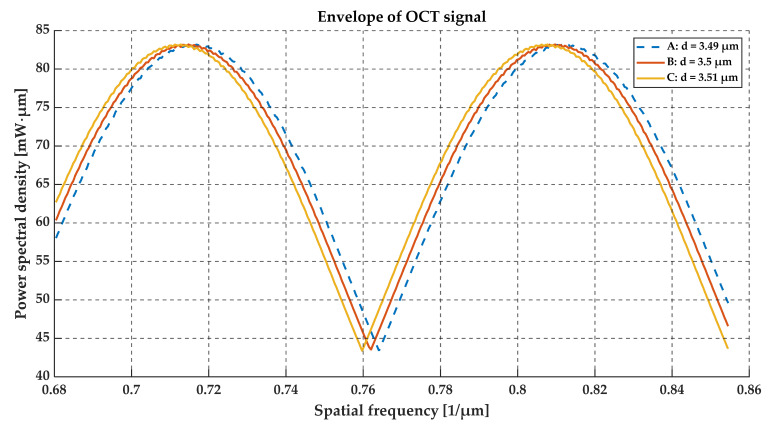
The envelope of the output OCT signal for the glass foils. The difference in the film thickness causes a noticeable shift in the position of the minimum value of the interference contrast.

**Figure 11:**
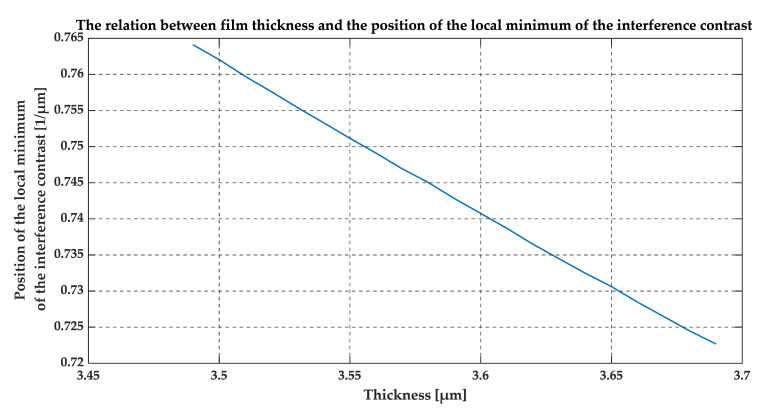
The relationship between the location of the minimum value of the interference contrast and the layer thickness of glass foil.

To assess the sensitivity of the OCT output signal to the film thickness, the optical signal at the output of the OCT system was calculated for glass foils with a refractive index of 1.5 and a thickness of 3.49 μm, 3.5 μm and 3.51 μm. The simulation results are shown in Figure 8, Figure 9, Figure 10 and Figure 11. We may observe that even small thickness changes result in notable changes in the output OCT signal. An analysis of the position of local minima of the interference contrast of the output OCT signal enables us to detect changes in the thickness over a range much lower than the axial resolution of the measurement system. For this purpose, the envelope of the calculated output OCT signal was extracted (to simplify the calculations the spectral characteristic of the light source was removed)—see Figure 10 and Figure 11. With an increase in thickness, the minimum of the envelope shifts into a longer wavelength and also a shorter wavenumber, which is shown in Figure 11.

## 3. Measurements

The experimental validation of the approach for simulating film properties using developed Equations (3), (9) and (10) were provided. Two experiments were carried out. All measurements were made with the use of the OCT system IVS-2000-PS (by Santec). The parameters of the system are summarized in Table 2.

### 3.1. Measurement of Thin Foil

**Figure 12:**
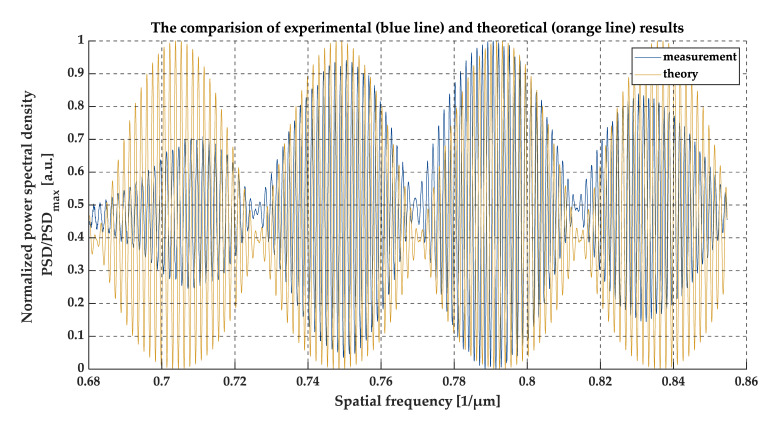
A comparison of experimental (blue line) with theoretical (orange line) results.

To verify the degree of matching the simulation and measurement results, a sample of known thickness and the refractive index was tested. The foil spacers intended for LCDs have stringent requirements to maintain their dimensions, especially thickness. Due to this, they are suitable samples for comparing the theoretical predictions with the measurement results. The thickness of the tested foil was 7 µm and the refractive index of the foil was 1.5862 at a wavelength of 1.320 µm (at a spatial frequency equal to 0.767 µm^−1^). The difference in the length of the optical paths in the Michelson interferometer was 0.7 mm, which also indicates the depth of the sample. A comparison between the theoretical simulation and the measurement data is shown in Figure 12. Moreover, the thickness was confirmed by measurements from two dial gauges. The accuracies of the dial gauges were 1 µm and 0.5 µm.

### 3.2. Measurement of the Wedge Cell

**Figure 13:**
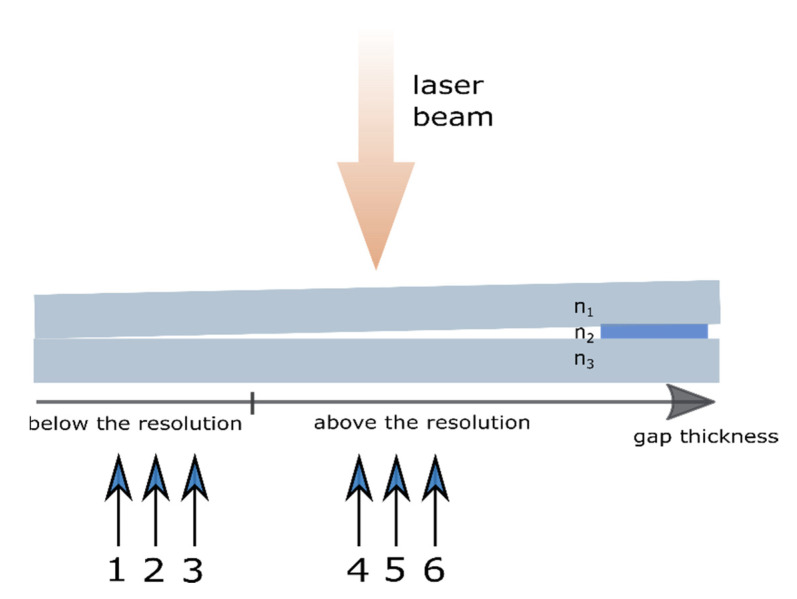
The theory and proof of concept of the wedge-cell measurement. The measurements were made at measurement points (P1–P6), where P1 is the thinnest point and P6 is the thickest point. According to the mathematical model of the layer, $n_{1}$ and $n_{3\;}$ are a refractive index of soda-lime-silica glass, and $n_{2\;}$ is a refractive index of air.

The wedge-cell experiment was carried out to confirm the capability of the OCT system for thin layer measurements. The methodology includes the thickness measurements of the air gap between two glass pieces for two cases. Firstly, the measurements were taken for a gap size below the OCT resolution. Next, the value of size was chosen above the resolution, however, the slight changes over the mean value were introduced to observe the differences in the recorded measurement signal. The device under test (DUT) consisted of two glass microscope slides made from soda-lime-silica glass with the standard dimensions of 76 × 26 × 1 mm^3^. Their surfaces were faced at one side and spaced by 125 µm at the other one. A diagram of the DUT is presented in Figure 13. The air gap thickness between the glass slides was tuned by moving the sample along the lateral direction under the measurement head of the OCT system. The ratio between gap size and the lateral shift is 1.64 µm per mm, which means that a 1-mm shift introduces a 1.64-µm change in gap thickness. In order to control the sample shift, the RB13D/M precise 3-axis translation stage (by Thorlabs, Inc., Newton, NJ, USA) was used. For each measurement, the raw data were pre-processed by applying signal filtering, measurement system dispersion compensation, and transformation from wavelength domain to wavenumber. The results are presented in the form of spatial frequency. These steps are the standard routine in signal processing in any OCT system.

**Figure 14:**
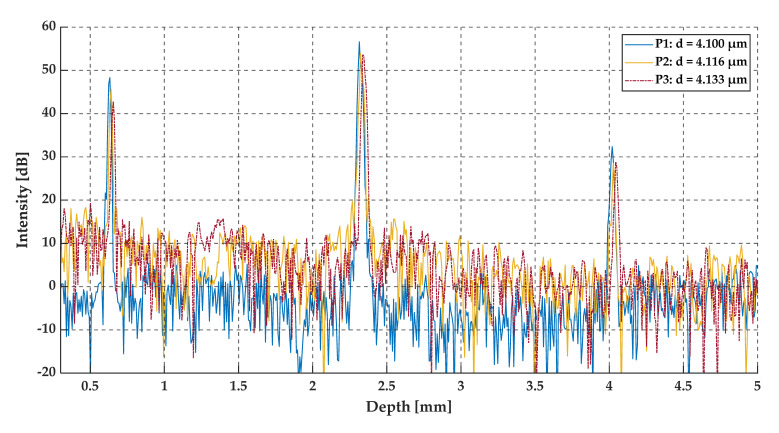
The A-scans at positions P1, P2, and P3.

**Figure 15:**
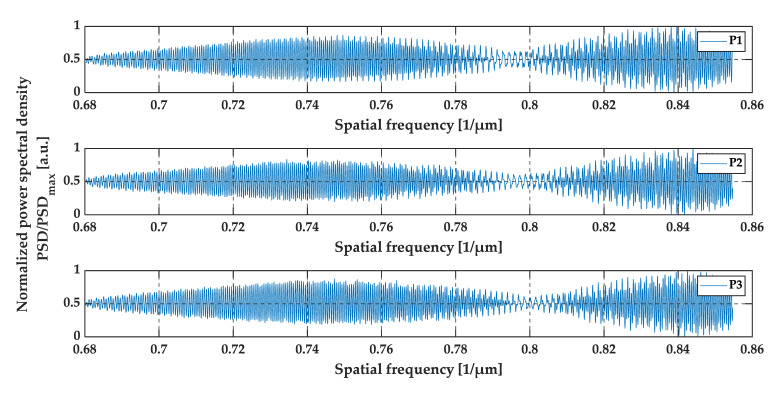
The preprocessed data at positions P1, P2, and P3.

**Figure 16:**
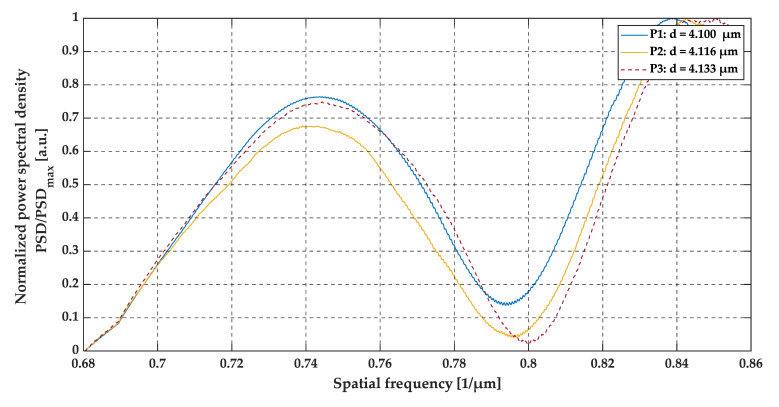
The envelopes of preprocessed data at positions P1, P2, and P3.

The measurements at positions P1, P2, and P3 (see Figure 13) were taken in a region where the value of gap thickness was below the resolution of the OCT system. The positions (the shift) of P1, P2, and P3, measured from the edge of the DUT, were 2.50 mm, 2.51 mm and 2.52 mm, which correspond to the gap thicknesses close to 4.100 µm, 4.116 µm and 4.133 µm, respectively. The A-scans made by the OCT system, the pre-processed data, and their envelopes are shown in Figure 14, Figure 15 and Figure 16.

The three peaks that may be observed in the A-scans (see Figure 14) correspond to the top glass surface, the intermediate layer (the air gap), and the bottom surface, respectively. All three A-scans overlap each other and there are no significant differences between these three measurements (at positions P1, P2, and P3). However, by analyzing the data presented in Figure 15 and Figure 16, the shift between the minima of oscillations may be observed. This is clearly expressed in the shift of the minima of the signals envelopes. For an air gap thickness of 4.100 µm, 4.116 µm and 4.133 µm the minima occurred at 0.794 µm^−1^, 0.796 µm^−1^ and 0.800 µm^−1^, respectively.

**Figure 17:**
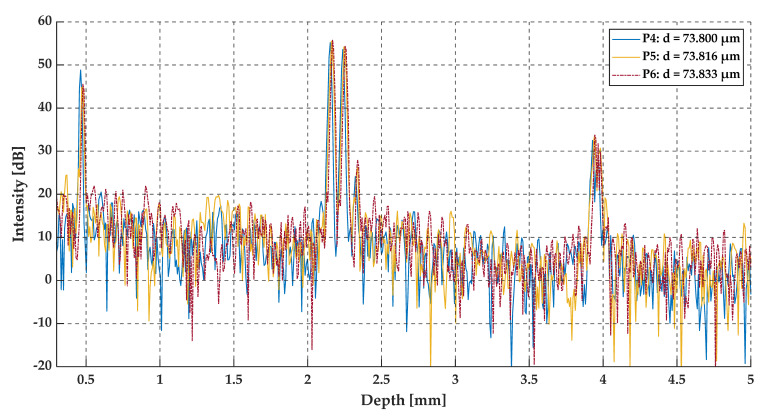
The A-scans at positions P4, P5, and P6.

**Figure 18:**
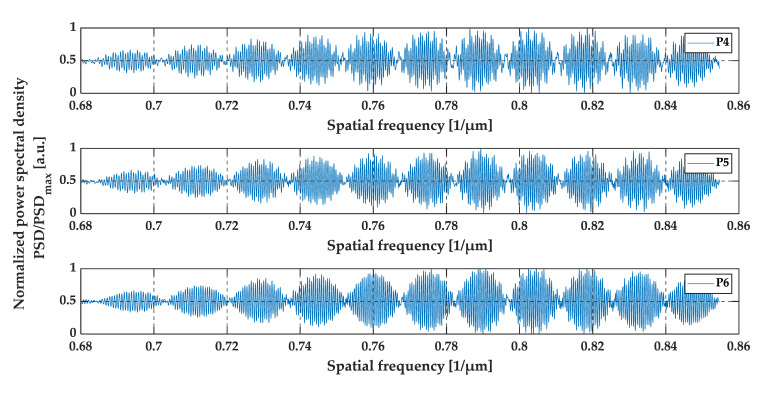
The preprocessed data at positions P4, P5, and P6.

**Figure 19:**
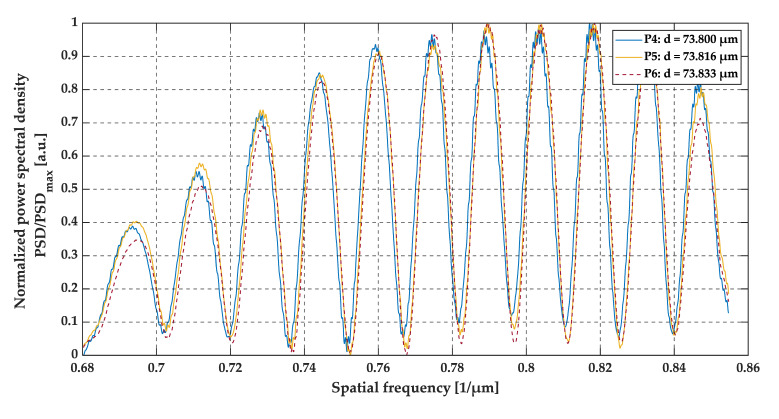
The envelopes of preprocessed data at positions P4, P5, and P6.

Positions P4, P5, and P6 were set at 45.00 mm, 45.01 mm and 45.02 mm from the edge of the sample, which corresponds to a gap thickness of 73.800 µm, 73.816 µm and 73.833 µm, respectively. The gap thickness at these positions was above the OCT resolution. Therefore, the gap appears at the two close recognized peaks in the middle for the A-scans. The A-scans, as well as the pre-processed data and their envelopes are presented in Figure 17, Figure 18 and Figure 19.

## 4. Discussion

### 4.1. General Overview of the Method and Proof of Concept

The analyzed signal includes three distinct components: the fast-changing amplitude modulated wave (carrier), the slow-changing amplitude modulated wave (envelope), and the spectral characteristics of the light source. Note that the desired sample parameters (thickness, refractive index, and location) are embedded within modulated waves. The envelope of the output OCT signal is correlated with the layer (or foil) thickness. The amount of local minima of the interference contrast in the spectrum depends closely on the layer thickness and refractive index of the layer. Thicker layers and layers with higher refractive index values entail a greater amount of local minima in the spectrum. The refractive index of the substrate and the medium in front of the layer affects modulation depth. The frequency of the carrier of the signal provides information about the depth of the layer. The higher frequency indicates the deeper location of the layer (see Section 2.2). The positions of the minima are related to the layer thickness. Simulations revealed that the minimum value occurs even for thin layers with a thickness under the resolution of the OCT system. The theoretical results show that the occurrence of the minimum value depends closely on the spectral range of the light source used. Due to this, it is possible to measure thicker layers using a setup with a broadband light source or by choosing an appropriate spectral range of light source. This analysis produces promising results to improve the resolution of the OCT method.

Simultaneously, the analyzed signal produces information about the location of the layer. The second component of the signal (carrier) is related to the depth or location of the layer. These data may be used to evaluate the thickness of a layer covered by another one, which is an unobtainable characteristic for such measuring techniques as profilometry. On the other hand, in the case of a layer with a known thickness, it is possible to calculate the refractive index or dispersion if it occurs.

### 4.2. Validation of the Method Implementation

The correctness of the method implementation was evaluated experimentally by performing OCT measurements of a sample that had well-defined parameters. For this test, an LCD inner spacer in the form of thin foil was chosen. The measurement data were compared with the simulation results. This was performed for the conditions defined by the optical and mechanical features of the sample under test. The experiment is described in detail in Section 3.1.

In order to the performance of the implementation, the number of minima (the minimum values of the envelopes), their positions, and the phase shift between simulated and experimentally obtained signals were analyzed. According to the experiment results, the positions of the minima of the amplitudes (envelopes) match together. It follows that the phase shift between both series of data is stable over the analyzed spectral range and close to zero. The observed differences in the amplitudes of the signals at the upper and lower part of the spatial frequency range may be explained by the shape of the spectral characteristics of the optical source, the optical features of the measurement system, and the tested sample. In conclusion, the simulation data are in close agreement with the measurement results, which positively verifies the performance of the implementation of the algorithm and method.

### 4.3. Thin Film Measurements with the Standard OCT System

The potential ability of the OCT system to estimate layer (or foil) thickness below the two-point OCT resolution defines the thesis of this research. This ability was confirmed by measuring the thickness of the wedge cell, which is described in Section 3.2. The performance of the cell allows for a precise control precisely the size of the gap between the inner surfaces by shifting the cell along the lateral direction. The ratio between gap size and the lateral shift is 1.64 µm per mm, which means that the 1-mm shift introduces a 1.64-µm change in gap thickness. During the experiment, a series of measurements in six different positions were collected. The measurements at positions P1–P3 were taken in the region where the overall gap thickness was below the OCT system resolution, while, the measurements at positions P4–P6 were made for the thickness above the resolution value.

From an analysis of the A-scans at positions P1–P3, it is difficult to estimate the real value of the gap size directly from the output OCT data. The gap is represented by only one single peak, which makes the differentiation of backscattered waves from the upper and lower interface of the gap impossible. Different conclusions may be drawn based on an analysis of the preprocessed data (see Figure 15) and their envelopes (see Figure 16). The shifts between the P1–P3 data series may be recognized. This may be clearly seen by observing the position of minima at the chart in Figure 16. Their values are equal to 0.794 µm^−1^, 0.796 µm^−1^ and 0.800 µm^−1^ at positions P1–P3, respectively. The shifts in the minima position in the spatial frequency domain were obtained for the gap thickness changes of the order of 16 nm. Referring to Figure 10, where the changes in the glass foil thickness were about 10 nm, a comparable shift in the position of envelope minima in spatial frequency occurs as at positions P1–P3 (see Figure 16). It follows that we are led to the conclusion that the mathematical modeling is correct and has performed well. Also, the potential of OCT for thin-film evaluation at the nanoscale level has been confirmed.

A slightly different case was analyzed at positions P4–P6. The gap thickness at these positions was above the two-point OCT system resolution, therefore, the two neighboring peaks in the middle of the A-scan were detected. Also, the number of minima at the preprocessed data plots (see Figure 18) increased. As previously mentioned, the differences in gap size at positions P4–P6 were of the order of ±16 nm, which cannot be seen at the A-scans (see Figure 17). All three A-scans overlapped each other almost perfectly. However, by analyzing the preprocessed data and their envelopes in the spatial frequency domain (see Figure 18 and Figure 19), the shift between each plot for positions P1–P3 may be seen. This shift corresponds to those slight changes in gap thickness, which also confirms the ability of OCT to track very small changes in film thickness, even those above the standard OCT resolution.

## 5. Conclusions

Mathematical considerations have been confirmed by experiments and indicate that it is possible to measure layer thickness by analyzing the location of the minima of the modulated depth of the obtained measurement output signal. Despite the simplicity of the proposed model, the simulated results are in close agreement with the experimental data for the cases considered. Furthermore, this method may be applied to any OCT system. Depending on the configuration of the measurement system, it is necessary to use an appropriate mathematical model of the interferometer used.

The proposed method may be a promising tool that can be used to provide more information about the tested layers than other techniques and improve the metrological potential of OCT measurements. Moreover, an analysis of the location of the minimum value of the interference contrast provides opportunities for further development, such as e.g., the application of optimization algorithms especially for multi-point measurements to assess layer morphology and layer thickness uniformity.

It should be noted that the OCT system used was not originally intended to perform layer thickness measurements below the two-point system resolution. For this purpose, advanced system calibration is required, which includes the dispersion of the OCT system, the dispersion of DUT, the power stability of the light source, and wavelength fluctuations. Only then, can the high accuracy and resolution of OCT measurements be achieved.

## Figures and Tables

**Table 1:** The conditions under which the simulations were carried out.

Item	Value
Beam intensity profile	Gaussian beam
Output power of the laser	10 mW
Central wavelength	1290 nm
Wavelength range	140 nm

**Table 2:** Parameters of the spectroscopic (S)-OCT system.

Item	Value
Light source type	20 kHz swept-source laser
Average output power	10 mW
Central wavelength	1290 nm
Wavelength range	140 nm
Axial resolution (in the air)	12 µm
Lateral resolution	15 µm
Frame rate	>4 fps
Max. depth imaging range/transverse imaging range	7 mm/10 mm
